# Sheep scab: comparison of spatial and temporal patterns determined by clinical diagnosis or ELISA

**DOI:** 10.1186/s13071-022-05564-5

**Published:** 2022-11-11

**Authors:** Chloe Makepeace, Emily Joanne Nixon, Stewart T. G. Burgess, Lesley Stubbings, Richard Wall

**Affiliations:** 1 School of Biological Sciences, University of Bristol, Bristol, BS8 1TQ UK; 2 Moredun Research Institute, Pentlands Science Park, Bush Loan, Penicuik, EH26 0PZ Midlothian UK; 3 LSSC Ltd, Oundle, PE8 4AL Northamptonshire UK

**Keywords:** Disease, ELISA, Mite, Outbreaks, Prevalence, *Psoroptes ovis*, Sheep scab

## Abstract

**Background:**

Ovine psoroptic mange (sheep scab) is an infectious condition caused by an allergen-induced hypersensitivity response to the mite *Psoroptes ovis*. Infestation results in clinical disease, economic loss and welfare issues in many sheep-producing countries. The aim of this study was to compare the prevalence and spatial pattern of sheep scab on contiguous farms, using both self-reported clinical outbreak history (2012–2020) and serological testing with an enzyme-linked immunosorbent assay (2021/2022).

**Methods:**

Farms included in the study were located in three regions of known high scab prevalence in North, Central and Southwest England. In total, 254 farms completed both a questionnaire, which provided the clinical scab history of the farm, and submitted results of serological testing with the ELISA.

**Results:**

A scab outbreak was reported by 17.4% (± confidence interval [CI]: 4.6%; *n* = 48) of farms in 2020 based on clinical diagnosis; scab was diagnosed by the ELISA on 25.6% (± 5.5%; *n* = 65) of farms in 2021/2022. Comparison of self-reported clinical scab cases with the ELISA test results identified a group of farms (*n* = 52) that did not report scab in 2020, or in some cases did not report having scab over the previous 8 years (*n* = 20), but whose flocks were nevertheless seropositive in 2021/2022.

**Conclusion:**

A small number of flocks, particularly those using common grazings in North England, where handling is infrequent, often comprising less susceptible sheep breeds, may have persistent scab infestations that are generally undetected by clinical inspection. The data highlight the advantages of serological testing to identify exposure to scab in flocks where clinical signs are less easily detected.

**Graphical Abstract:**

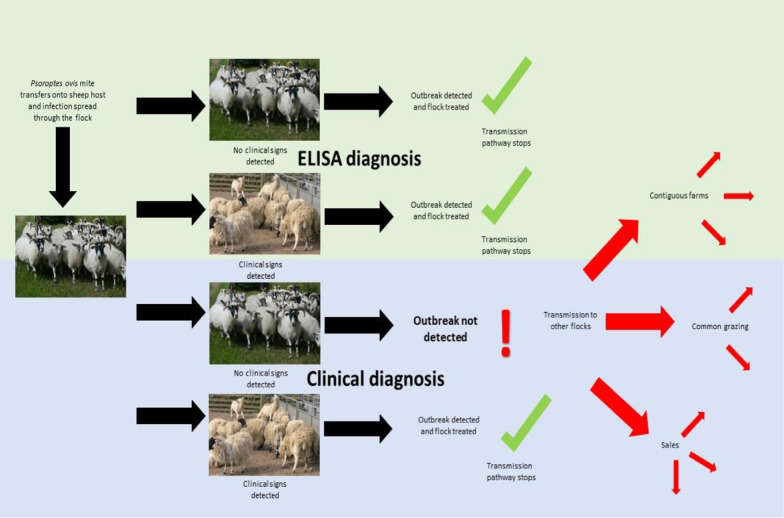

## Background

Ovine psoroptic mange (sheep scab) is an infectious condition caused by an allergen-induced hypersensitivity response to the mite *Psoroptes ovis* [1, 2] Infestation results in clinical disease, economic losses and welfare issues in most sheep-producing countries. Sheep scab was reintroduced into the UK in 1972 following a 20-year period of apparent eradication [3]. Over the subsequent 20 years, there were numerous unsuccessful attempts at re-eradication through mandatory treatment programmes [4, 5]. However, eventually the control of sheep scab was deregulated in 1992, and this was followed by a 60-fold increase in the number of outbreaks from 1992 to 2007 [4]. The most recent national estimates indicate that there are about 8000–10,000 outbreaks per year [6, 7], resulting in an estimated cost of £78–202 million annually to the sheep industry in the UK [8]. Although current acaricidal treatments are generally effective at controlling outbreaks, there is confirmed resistance to all three macrocyclic lactones (ivermectin, doramectin and moxidectin) that are available for treatment [9], although the prevalence of resistance to these acaricides is unknown at present. Organophosphate (diazinon) dip remains the only acaricide available for treatment without reported resistance [5], but increased resistance may be anticipated in the future as scab control becomes more dependent on its use, unless the procedure is used responsibly. Widespread resistance, in the absence of the development of new acaricides, is likely to result in a considerably higher scab prevalence in the UK. As a result, the development of precision management strategies based on a better understanding of the causes of scab transmission is essential.

The use of effective diagnostic testing for sheep scab would help to reduce unnecessary treatments. Generally, sheep scab diagnosis has been by clinical examination and skin scrapes for a confirmatory diagnosis via microscopy. These methods have low sensitivity, with detection possibly being as low as 18% in asymptomatic carriers [10]. Although diagnosing animals with cryptic or pre-clinical infections can be difficult, these animals may still transmit the infection [2]. In natural infestations, the sub-clinical phase ranges from several weeks to months [10]. This carrier group of animals presents a challenge for control and represents a major contributor to the transmission to naive flocks. However, a new enzyme-linked immunosorbent assay (ELISA) [11] provides an essential diagnostic tool for identifying sub-clinical sheep scab through serological testing using the Pso o 2 antigen [12].

A previous modelling study investigated the spatial and temporal dynamics of scab at a national scale and identified geographic regions in the UK where scab prevalence is higher than the national average. These regions are associated with areas of high sheep farm density and movements [13], as well as with specific farming practices, such as the use of shared (common) grazings [7]. Elevation, temperature and precipitation are significant predictors of scab outbreaks, particular when determining risk in uplands regions; in lowland regions, the density of sheep farms appears to be a better predictor of risk [7]. Southwest England (lowlands) has warm summers with mild winters, North England (uplands) has cool summers with mild to cold winters and the Midlands is characterised by a transitional climate that falls in between; all regions have year-round rain.

Within these regions of high scab prevalence, a small number of farms experience repeated uncontrolled outbreaks, and farms with contiguous scab-infested neighbours are more likely to have scab than those without [14]. Beyond the boundaries of these regions, scab prevalence is lower, and it has been argued that lower farm densities result in lower transmission by sheep-to-sheep contact or environmental contamination and, in the absence of long-distance movements, scab transmission would be expected to self-limit spatially [13]. Farm-to-farm transmission through contact with infected animals from contiguous neighbouring farms might be expected to lead to localised spatial patterning of outbreaks within high-risk areas. Understanding the generality of this scenario is important because it would allow scab management to be directed towards the areas at highest risk and the farms that function as reservoirs of persistent infection.

The aim of the study presented here, therefore, was to compare the prevalence and spatial pattern of scab outbreaks in groups of contiguous farms in three regions of high scab prevalence in England, recruited as part of a regional control project funded by the Rural Development Programme for England (RDPE). Scab prevalence was determined using both clinical outbreak history and serological ELISA testing results; the results were used to examine localised outbreak patterns that could help demonstrate the role of farm-to-farm scab transmission.

## Methods

### Scab history

Sheep farms were recruited in three regions that had previously been identified as having a higher than average prevalence of sheep scab [6, 7, 13]: these were the North (Cumbria, Lancashire, North Yorkshire and Northumberland), the Midlands (Shropshire and Herefordshire) and the Southwest (Cornwall and Devon). Between 70 and 100 farms in each region were recruited as part of a single large cluster or multiple smaller ones. A cluster was defined as a group of farms that were contiguous (< 0.5 km between boundaries) and/or shared common grazings. Where possible, the physical boundaries of a cluster were defined by roads, rivers or woodlands. The data for the current study were collected as part of a wider project to examine the effectiveness of a coordinated treatment approach for sheep scab control in these areas, which will be reported elsewhere.

A retrospective questionnaire, describing the history of sheep scab on each farm, was completed by each farm/farmer in late 2021 during a face-to-face meeting or by telephone appointment with a regional coordinator. Farmers were asked about their experience with scab outbreaks in 2020 and in the previous years back to 2012, as well as for detailed information on their location, farm, flock and management practices. Respondents from 276 farms (North = 83, Midlands = 93, Southwest = 100) completed the questionnaire in sufficient detail to allow them to be included in the analysis. The data from completed surveys were entered into an Excel spreadsheet (Microsoft Inc., Redmond, WA, USA). Not all farmers answered every question, but where the data allowed, incomplete questionnaires were included in the analysis, and, as a result, denominators were not consistent between questions.

### Enzyme-linked immunosorbent assay

The serological test (ELISA) is applied at a flock or group level and requires a minimum of 12 animals to be tested as a representative sample [15]. For this study, blood testing of 12 sheep per farm began in June 2021, and the cut-off date for blood tests used in this analysis was 31 March 2022. Among the farms recruited, there were 254 (North = 83, Midlands = 80, Southwest = 91) where at least one blood test had been performed before the cut-off date and the questionnaire had been completely sufficiently for use in our analysis. The blood samples were processed (Biobest Laboratories, Edinburgh, UK) following a standard commercial ELISA protocol [11]. Plates were read at 450 nm on a microplate reader to obtain optical density (OD) values, and these were reported as OD_450_ × 100. The flock was determined to be positive or negative for scab using a Bayesian hierarchical algorithm (Giles Innocent, personal communication). The recommended sample size of 12 animals had been designed to achieve a flock-based sensitivity and specificity of approximately 95%, assuming that the within-flock prevalence of sheep scab would be 20% at the time of testing. The posterior probability was assessed on a scale of 0 to 1, and values > 0.5 were assumed to be positive for sheep scab in this analysis. Some farms submitted multiple blood samples for ELISA serological testing if the previous results were inconclusive or unexpected or when testing multiple management groups. In these instances, a single positive result was considered sufficient to designate a farm as positive.

### Data analysis

All data analyses, based on the reported occurrence of clinical scab in 2020, the previous 8 years and ELISA-determined presence in 2021/2022, were conducted using R version 4.1.1 [16]. Continuous variables were analysed with univariate regression analysis using the *glm* function in R with "family = binomial”. For categoric variables, Chi-squared (*χ*) analysis, using the *chisq.test* function in R, was used to compare subsets. Sequential Bonferroni corrections were made to the acceptance thresholds (*P-*value) when multiple comparisons were made.

### Spatial analysis

Farms were provided with a postcode or Ordinance Survey (OS) map reference number, which was converted to British national grid (BNG) coordinates (Easting and Northing) for use in spatial analysis [17]. Spatial analysis was conducted separately for each region. A shapefile of the counties making up each study region was imported into R using the *readOGR* function from the *rgdal* R package. The spatial area was then reduced to the area containing the points using the *crop* function from the *raste*r R package. These returned a geographic shapefile subset containing just the area containing the farms. To confirm that the underlying distribution of the farms deviated from complete spatial randomness (CSR), the G-function and significant envelope (with 100 Monte Carlo simulation repeats) were calculated using the *Gest* and *envelope* functions from the *spatstat* R package.

The relative risk of cases (farms that reported an outbreak/tested positive with the ELISA) and controls (farms that did not report an outbreak/tested negative with the ELISA) was analysed using the *risk* function from the *sparr* R package. The bandwidth (smoothing parameter) had a pooled symmetry, meaning the kernel size was equal when conducting the case and control analysis, and was adaptive, whereby the smoothing of the relative risk surface was equal for both the case and control risk analysis [18, 19], accounting for the heterogeneous underlying distribution of the points [20]. Diggle edge correction was applied [21] to account for points near the edge of the study regions that may have non-surveyed neighbouring points not included in the analysis. The risk surface was plotted, and significance contours overlayed using the *tol.contour* function from the *sparr* R package. A SatScan [22] was used to determine the locations and significance of the spatial clustering. This automatically accounts for the underlying distribution of the data points. A Bernoulli model was used as the points were binary (i.e. 0 [control] or 1 [case]). To understand the extent of possible direct transfer between contiguous farms, the number of outbreak farms with outbreak contiguous neighbouring farms was calculated. A distance matrix calculating the Euclidean distance between all points was created using the *dist* function from the *stats* R package. Farms located < 2000 m between centre points were deemed to be contiguous neighbours for this analysis; this standardised figure, previously used by Nixon et al. [13], represents the radius of an average farm in the UK and is a distance within which it is assumed that direct transmission through the environment may be likely. The percentage of outbreak farms with at least one outbreak contiguous neighbouring farm was calculated.

## Results

### Scab prevalence based on clinical diagnosis

In 2020, 17.4% (± confidence interval [CI] 4.6%; *n* = 48) of the farms in the study reported a scab outbreak. There was a highly significant difference in scab prevalence between the three regions in 2020 (*χ*^2^ = 13.6,* df* = 2, *P* = 0.001), with prevalence highest in the Midlands (26.9% ± 10.5%; *n* = 25), followed by the North (19.3% ± 9%; *n* = 16) and lowest in the Southwest (7.0% ± 5.6%; *n* = 7) (Table 1). Between 2012 and 2019, at least one scab outbreak was reported at 34.4% (± 5.7%; *n* = 95) of farms across all regions. There was a significant regional difference (*χ*^2^ = 13.5,* df* = 2, *P* = 0.001), with the number of farms with reports of scab between 2012 and 2019 being highest in the Midlands (45.2% ± 10.1%; *n* = 42), followed by the Southwest (37% ± 9.5%; *n* = 37) and lowest in the North (19.2 ± 8.5%; *n* = 16).

**Table 1:** The scab status of farms (*n* = 248) as self-reported in 2020, assessed by observation of clinical signs, and in 2021/2022 when exposure to scab was determined by serological testing using the enzyme-linked immunosorbent assay

Scab status		Number of farms^a^						
Clinical signs (2020)	ELISA (2021/2022)	All	Midlands		North		Southwest	
			Other	Common grazers	Other	Common grazers	Other	Common grazers
Positive	Positive	13	5	1	3	2	2	0
Positive	Negative	30	12	3	7	3	4	1
Negative	Positive	52	10	1	8	15	13	5
Negative	Negative	153	41	5	17	26	54	10

*ELISA* Enzyme-linked immunosorbent assay

^a^The number of farms are also broken down into region (Midlands, North and Southwest) and by whether farmers used common grazing or other grazing system

**Fig. 1:**
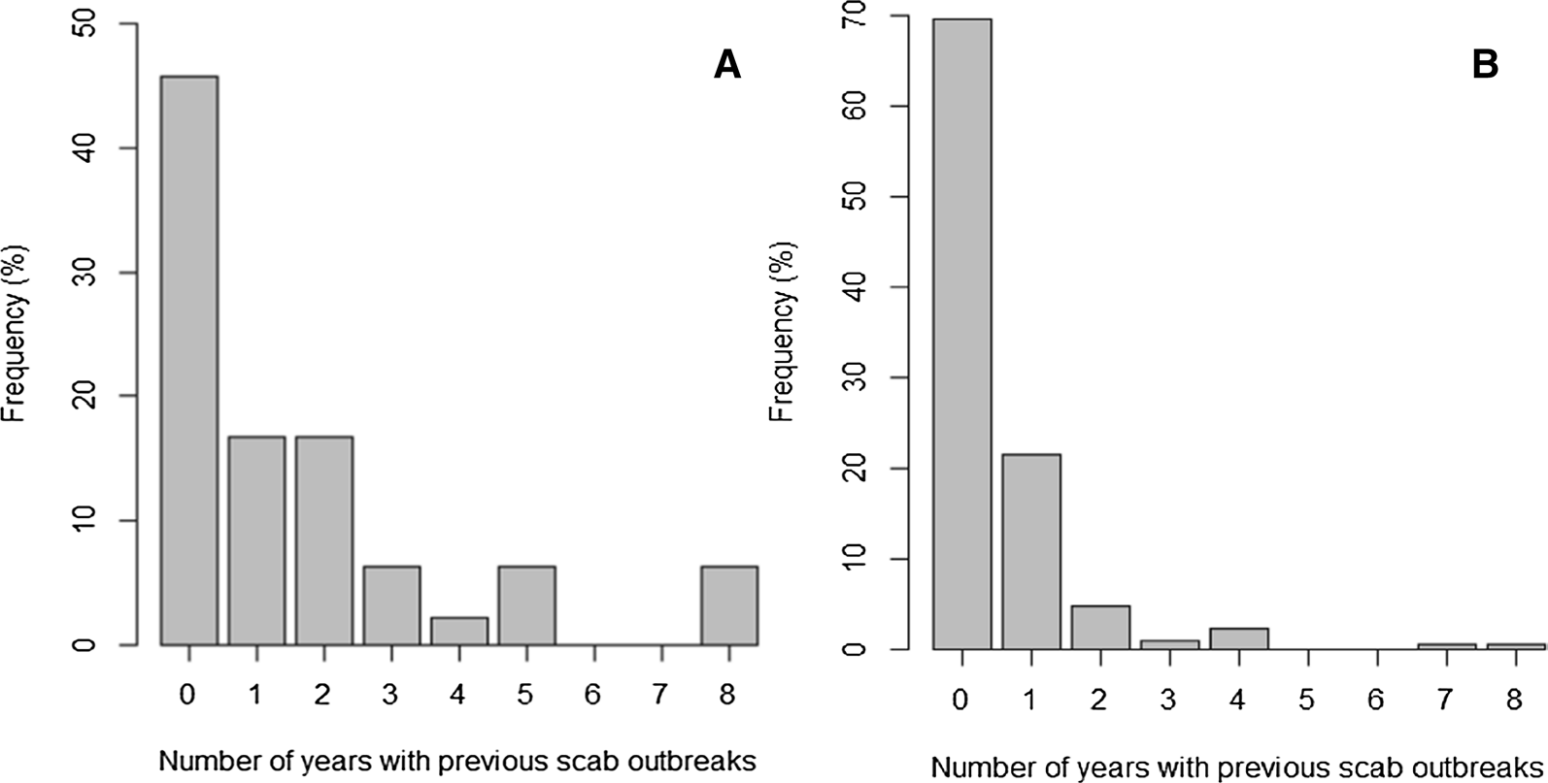
**a** The number of years a farm had experienced scab outbreaks in the previous 8 years (2012–2019) on farms which did report a scab outbreak in 2020 (*n* = 48). **b** The number of years a farm had experienced scab outbreaks in the previous 8 (2012–2019) on farms which did not report a scab outbreak in 2020 (*n* = 228)

**Fig. 2:**
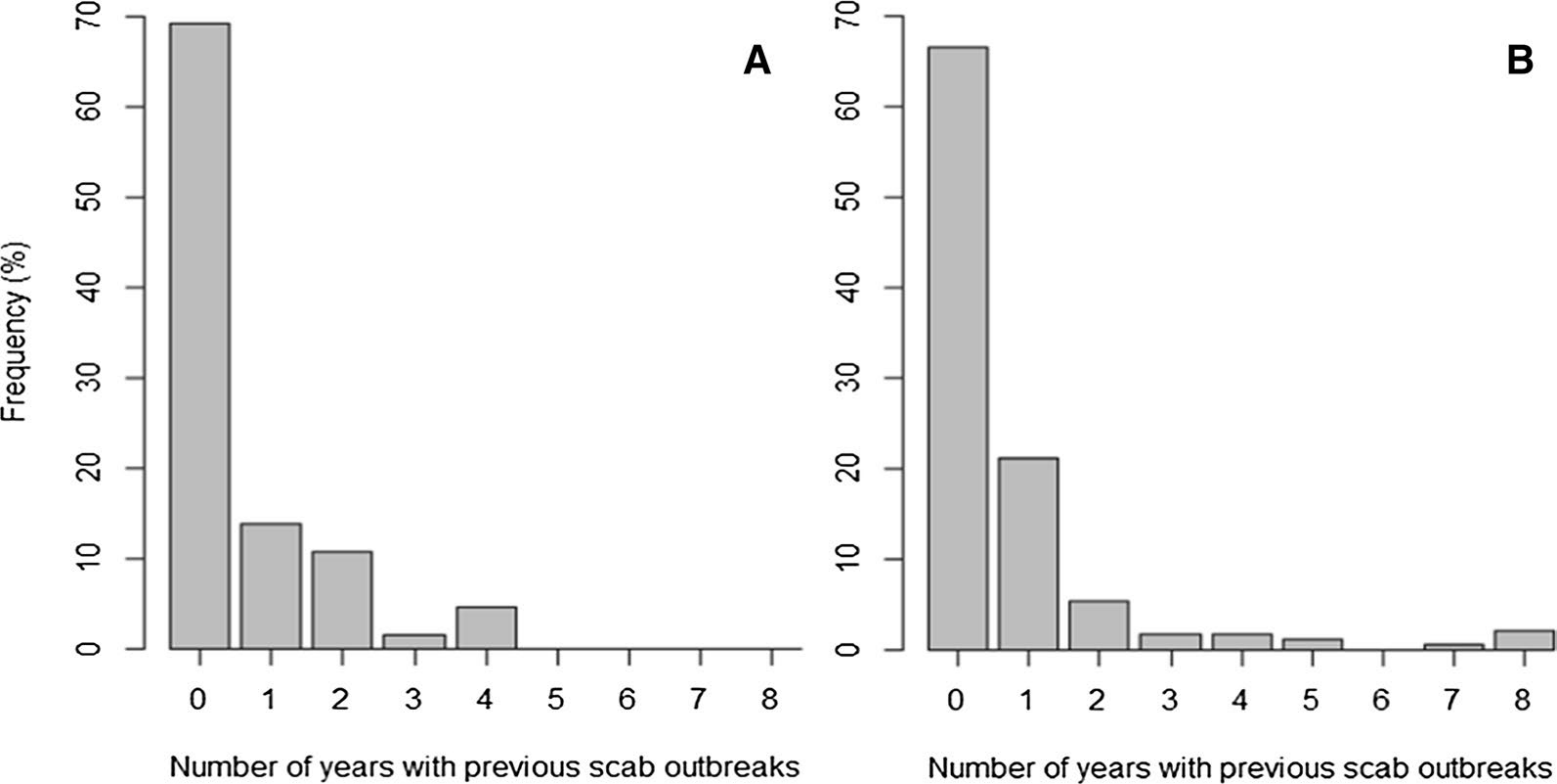
**a** The number of years a farm had experienced scab outbreaks in the previous 8 years (2012–2019) on farms which had a positive enzyme-linked immunosorbent assay (ELISA) result in 2021/2022 (*n* = 65). **b** The number of years a farm had experienced scab outbreaks in the previous 8 (2012–2019) on farms which had a negative ELISA in 2021/2022 (*n* = 189)

Four farms reported an outbreak of scab every year since 2012 (Fig. 2a). Of the farms reporting a scab outbreak in 2020, 54.2% (± 14.5%; *n* = 26) also reported having experienced at least one scab outbreak in the previous 8 years (Fig. 1a). Farmers that reported having had at least one outbreak in the previous 8 years were significantly more likely to have reported a scab outbreak in 2020 (*χ*^2^ = 9,* df* = 1, *P* = 0.002; Fig. 1a). The number of years in which a scab outbreak had been previously reported for a farm significantly increased the likelihood of an outbreak being reported in 2020 (coefficient: 0.426, standard error [SE]: 0.106, *P* = 0.001). Farms that reported at least 1 year with an outbreak in the previous 8 years were 2.72-fold more likely to report an outbreak in 2020. Farms that reported an outbreak in 2019 were 8.61-fold more likely to report an outbreak in 2020. Of the 228 farms that did not have a scab outbreak in 2020, 69.7% (± 6.1; *n* = 159) reported not having had a scab outbreak in the previous 8 years, while 59.2% (± 6.6%; *n* = 135) reported never having experienced a scab outbreak (Fig. 2b).

### Scab prevalence based on serological testing

Serological positives were determined on 25.6% (± 5.5%; *n* = 65) of farms. There was no significant difference in regional prevalence (*χ*^2^ = 4.3,* df* = 2, *P* = 0.12). Prevalence was highest in the North (33.7% ± 10.5%; *n* = 28), followed by the Southwest (22% ± 8.9%; *n* = 20) and the Midlands (21.2% ± 9.4%; *n* = 17). Overall, significantly more scab outbreaks were detected using the ELISA in 2021/2022 compared to self-reported clinical cases in 2020 (*χ*^2^ = 4.8,* df* = 1, *P* = 0.03). The number of outbreaks reported was significantly greater in the North (*χ*^2^ = 3.7,* df* = 1, *P* = 0.05) and the Southwest (*χ*^2^ = 7.6,* df* = 1, *P* = 0.005). There was no significant difference in the number reported between 2020 and 2021/2022 in the Midlands (*χ*^2^ = 0.5,* df* = 1, *P* = 0.5).

Farms that reported having had at least one outbreak in the previous 8 years were not more likely to report a positive ELISA test (*χ*^2^ = 0.05,* df* = 1, *P* = 0.82). A total of 65 farms had a positive ELISA result, with the majority (69.2% ± 11.2%, *n* = 45) reporting that they had not had a scab outbreak in the last 8 years (Fig. 2a). A negative ELISA result was reported at 189 farms, with 66.7% (± 6.7; *n* = 126) reporting that they had not had a scab outbreak in the previous 8 years (Fig. 2b).

Self-reported scab outbreaks in 2020 on individual farms were compared to the ELISA results from 2021/2022 (Table 1). Most farms who had no self-reported outbreaks in 2020 had negative ELISA results in 2021/2022 (62.2% ± 6.1%;* n* = 158). A relatively small number of farms with a self-reported outbreak in 2020 tested positive with the ELISA in 2021/2022 (5.1% ± 3%; *n* = 13). Overall, in terms of scab testing, with the ELISA, more farms changed from negative to positive (20.5% ± 5%; *n* = 52), than from positive to negative (12.2% ± 4.2%, *n* = 31). Of the 52 farms that appeared to be negative for scab in 2020 based on clinical signs but were serologically positive in 2021/2022, there was a difference between the regions, with significantly more than expected in the North than in the Midlands (*χ*^2^ = 3.9,* df* = 1, *P* = 0.05). Also, in the North, twice as many farms which changed status from no reported outbreak in 2020 to being positive for scab in 2021/2022 as determined by ELISA were those that used common grazings (Table 1), a pattern not seen in the other regions.

### Spatial distribution of outbreaks

Based on the self-reported 2020 outbreak data, 38.4% (± 5.7%; *n* = 106) of farms had a contiguous neighbouring farm which reported having a scab outbreak, but only 18.9% (± 7.5%; *n* = 20) of these reported having an outbreak themselves. Of the 48 farms that reported a scab outbreak in 2020, 41.7% (± 13.9%; *n* = 20) had at least one contiguous neighbouring farm that also reported an outbreak. Farms with outbreaks in the Midlands were significantly more likely to have a neighbouring farm that also reported an outbreak (64% ± 19.8%, *n* = 16) than their counterparts in the North (25% ± 23.1%, *n* = 4) or Southwest regions (*n* = 0) (*P* = 0.072). Overall, having a contiguous neighbouring farm with an outbreak did not significantly change the likelihood of a farm reporting a scab outbreak in 2020 (*χ*^2^ = 0.12,* df* = 1, *P* = 0.73).

As anticipated, the underlying distribution of farms across the study areas were significantly different from random (as determined by the G-envelope function analyses). There were no areas of significantly elevated risk within any of the three regions, as identified by kernel density relative risk estimation. This was supported by SatScan, which did not identify any significant clusters within each region. L-function case–control comparison in the North and Southwest regions showed that case points had similar clustering to control points. However, in the Midlands, cases were more clustered than controls at a radius of < 1.5 km.

Based on the 2021/2022 ELISA serological data, kernel density and SatScan analyses were similarly unable to identify any significant clustering. However, in the L-function case–control comparison, the Southwest and Midlands cases were more clustered than controls at a radius of < 4.5 km and < 3 km, respectively. Mirroring the 2020 analysis, there was no significant relationship between contiguous neighbouring farms and the likelihood to report an outbreak using the ELISA (*χ*^2^ = 1.75,* df* = 2, *P* = 0.42). Just under half (46.9% ± 6.1%; *n* = 120) of farms had contiguous neighbouring farms with a positive ELISA result, but only 18.3% (± 6.9%; *n* = 22) of these themselves had a positive ELISA result. The number of positive farms with a positive contiguous neighbouring farm was highest in the Midlands (41.2% ± 25.3%; *n* = 7) and the Southwest (45%% ± 23.2%; *n* = 9), but much lower in the North (21.4% ± 11.6%; *n* = 6).

## Discussion

The present study examined the temporal and spatial patterns of sheep scab in three high-risk regions in England, as self-reported by farmers based on clinical signs, and then compared the patterns with the results from ELISA seroprevalence recorded in 2021/2022. The three regions included in the study were selected because of their relatively high prevalence of scab, as detected in previous studies [6, 14]. Prevalence was highest in the North, followed by the Southwest and the Midlands by both the clinical observation and ELISA. This pattern may be linked to the climatic conditions, sheep breeds and husbandry practices specific to each region.

Elevation, temperature and precipitation are significant predictors of scab outbreaks, particularly in more northerly upland regions, whereas in more southerly lowland regions, sheep density is a better predictor of risk [7].

Notably, when considering self-reported scab, even in the three areas included in the study, most farms were negative in 2020 and had been consistently negative over the previous 8 years. In contrast, a small number of farmers self-reported persistent scab outbreaks over the same period. This supports the conclusions of Rose et al. [7] that there is a small a group of farms which act as persistent foci of unresolved scab.

Comparison of self-reported clinical scab patterns with the ELISA test results identified four groups of farms. The largest group comprised farms that had never reported scab, did not report scab in 2020 and were also seronegative in 2021/2022. The smallest group were farms that reported clinical scab in 2020 and were seropositive in 2021/2022, having presumably failed to treat the outbreak, or the cause of the outbreak, successfully. A third group were farms that had reported scab the previous year, but had a negative result for the ELISA in 2021/2022. This group may reflect successful treatment, or pruritic sheep may previously have been misdiagnosed with scab rather than louse infestation. Alternatively, because on some farms flocks are divided into different management groups, and farmers and veterinarians were asked to self-select 12 sheep for testing with the ELISA, the animals selected may not have been representative of the entire flock.

Of particular interest is the fourth group of farms, namely those that did not report scab in 2020 but which were seropositive in 2021/2022 (20.5% ± 5%; *n* = 52); in some cases, these were farms that had not reported a clinical case of scab over the previous 8 years (30.8% ± 11.7%; *n* = 20). It is notable that the majority of farms in this group were located in the North and were predominantly flocks that used common grazings. A likely explanation is that scab had been consistently undiagnosed in these flocks: hill breeds of sheep may show fewer clinical signs than lowland breeds [23] and sheep grazing commons may only be inspected closely when sheep are periodically gathered. Hence, scab infestations may have persisted in these flocks undetected for a prolonged period. This is a critical point because sheep from these flocks may be sold or moved around the country and act as carriers for scab infestation into lowland areas.

Self-diagnosis of scab and the subsequent use of retrospective questionnaires to gather prevalence data are critically dependent on the accuracy with which farmers diagnose, recollect and then are willing to report scab outbreaks. The data collected in the present study suggest that the use of serological testing, rather than relying on clinical signs and skin scrapings, allows the identification of farms where scab is likely to have been present but where farmers had not previously thought that there was a problem.

Aggregated patterns in scab outbreaks at a national and regional scale have been identified previously [13, 14]. Analysis of reported outbreaks between 1973 and 1992 found highly significant space–time clustering, with very local spread (< 12 km) within 5 months of an outbreak [4]. To explain these patterns, it has been argued that sheep-to-sheep and environmental infection are likely to be important local transmission routes within clusters of highly aggregated farms [13]. As a result, in the present study, a pattern in the spatial distribution of scab outbreaks might have been expected. However, cluster analysis using comparisons of case–control L functions found little evidence of any pattern with the data from either diagnosis method. It is likely that the complexity of land use patterns at an individual farm scale makes the detection of such localised patterns difficult to quantify [24]. Given the mosaic pattern of land use by individual farmers, a description of a farm and its neighbouring farms based on a single central point is likely to be a gross oversimplification and hides the complexity of the movement of sheep around the fields grazed by an individual farmer. Additionally, the movement of sheep between fields may bring these sheep into contact with animals from non-contiguous neighbours.

The data demonstrate the value of the ELISA as a screening tool, particularly in identifying and treating sub-clinical/cryptic outbreaks. However, if individual farmers are required to pay for the costs associated with blood sampling and the ELISA test, it is likely that this testing route will only be used by farmers in high-risk areas or by farmers who think they have an outbreak [25]. As shown here, farmers may be unaware of chronic infestation, particularly in breeds that show less pronounced clinical signs and are more extensively grazed. Hence, if the ELISA is to be integrated into an extensive control programme it is likely that this will need to be centrally funded and co-ordinated into a regional management programme.

## Conclusion

There were significantly more scab outbreaks detected in the flocks studied using the ELISA in 2021/2022 compared to the self-reported clinical cases in 2020. Scab was not reported for a small number of flocks in 2020, but which were seropositive in 2021/2022 with the ELISA. These primarily consisted of those farms using common grazings in North England, where handling is infrequent, and where there are often less susceptible sheep breeds; these may have persistent scab infestations that, generally, remain undetected by clinical inspection. The data highlight the advantages of serological testing to identify exposure to scab in flocks where clinical signs are less easily detected.

## Data Availability

The datasets generated during the current study are not publicly available in accordance with the data protection act.
